# Lack of effect of intermittent preventive treatment for malaria in pregnancy and intense drug resistance in western Uganda

**DOI:** 10.1186/s12936-015-0909-7

**Published:** 2015-09-26

**Authors:** Vera Braun, Eva Rempis, Alexandra Schnack, Sarah Decker, John Rubaihayo, Nazarius Mbona Tumwesigye, Stefanie Theuring, Gundel Harms, Priscilla Busingye, Frank P. Mockenhaupt

**Affiliations:** Institute of Tropical Medicine and International Health, Charité-University Medicine Berlin, Berlin, Germany; Public Health Department, Mountains of the Moon University, Fort Portal, Uganda; School of Public Health, College of Health Sciences, Makerere University, Kampala, Uganda; Holy Family Virika Hospital, Fort Portal, Uganda

## Abstract

**Background:**

Intermittent preventive treatment in pregnancy (IPTp) with sulfadoxine–pyrimethamine (SP) is widely implemented in sub-Saharan Africa for the prevention of malaria in pregnancy and adverse birth outcomes. However, in areas of intense SP resistance, the efficacy of IPTp may be compromised.

**Methods:**

A cross-sectional study among 915 delivering women (728 analysable live singleton deliveries) was conducted in Fort Portal, western Uganda, to assess associations of reported IPTp use, *Plasmodium falciparum* infection, maternal anaemia, low birth weight, and preterm delivery, and to estimate the degree of SP resistance as reflected by *pfdhfr/pfdhps* mutations.

**Results:**

*Plasmodium falciparum* infection was detected by PCR in 8.9 % and by microscopy of placental blood samples in 4.0 %. Infection was significantly associated with stillbirth, early neonatal death, anaemia, low birth weight, and pre-term delivery. Eighty percent of the women had taken at least one dose of IPTp, and more than half had taken two doses. As compared to women without chemoprophylaxis against malaria, IPTp had no significant influence on the presence of *P. falciparum* infection (13.8 vs. 9.6 %, *P* = 0.31). Nor was it associated with reductions in anaemia, low birth weight or preterm delivery. *P. falciparum* with intense SP resistance (*pfdhfr/pfdhps* quintuple or sextuple mutations) were observed in 93 % (*pfdhps* 581G, 36 %), and the additional high resistance allele *pfhdr* 164L in 36 %.

**Conclusions:**

In Fort Portal, Uganda, reported use of IPTp with SP does not provide an observable benefit. The molecular markers of *P. falciparum* indicate high grade SP resistance reaching the threshold set by WHO for the discontinuation of IPTp with SP. Alternative approaches for the prevention of malaria in pregnancy are urgently needed.

## Background

Despite the implementation of intermittent preventive treatment in pregnancy (IPTp) with sulfadoxine–pyrimethamine (SP) in sub-Saharan Africa starting more than two decades ago, malaria in pregnancy continues to be a major public health problem. Pregnant women form a specific risk group for *Plasmodium falciparum* infection, malaria and related consequences, which include abortion, stillbirth, maternal anaemia, low birth weight (LBW), preterm delivery, and, annually, up to 200,000 infant deaths [1]. In highly endemic regions, primiparae are at particular risk due the lack of specific immunity preventing the placental sequestration of pregnancy-specific *P. falciparum* strains. Placental sequestration gives rise to local inflammation and also may result in placental infection in the absence of detectable peripheral blood infection [2–4].

Coverage with IPTp is low (<25 %) in African countries with an IPTp policy [5]. Moreover, the effectiveness of IPTp critically depends on parasite sensitivity to the drug, but SP resistance of *P. falciparum* has spread across Africa and intensified particularly in the East of the continent [6, 7]. In 2012, WHO modified the recommendation of two doses of IPTp with SP during pregnancy in areas of moderate to high transmission towards administration at each scheduled antenatal care (ANC) visit but at least 1 month apart [8]. This accords with the observation of less malaria and better birth outcomes using three or more doses of SP as compared to the standard two-dose regimen [9]. While the effectiveness of this approach has yet to be proven in areas of intense resistance, data from East Africa suggest at least partial failure of IPTp with SP in improving overall pregnancy outcomes [10–13]. In addition, in areas of intense SP resistance in Tanzania, IPTp among infected women was associated with increased placental parasite density and inflammation [14] as well as an increased risk of severe malaria in the offspring [15], and infections with highly resistant parasites were associated with lower birth weight [16].

Resistance to SP is conferred by mutations in the *P. falciparum* dihydrofolate reductase (*pfdhfr*) and dihydropteroate synthase (*pfdhps*) genes: a triple mutation of *pfdhfr* (108N -5I1-59R) combined with *pfdhps* mutations 437G and 540E (*pfdhfr/pfdhps* quintuple mutant) is predictive for SP treatment failure in children, even more so in case of an additional *pfdhps* 581 mutation (sextuple mutant). *pfdhfr* 164L is linked with high grade SP resistance [6, 17–19]. Recent work has shown that the effectiveness of IPTp declines with an increasing population prevalence of the *pfdhps* 540E mutation (representing the *pfdhfr/pfdhps* quintuple mutation) even though some effect on birth weight remains even at very high prevalence. Increasingly, the *pfdhfr/pfdhps* sextuple mutation including the *pfdhps* 581 variant is considered an informative marker on whether IPTp might be compromised or not [20, 21]. In line with that, a recent study from Malawi reported failure of parasite suppression by IPTp in the presence of sextuple-mutant parasites [22].

In Uganda, policy recommendation is at least two doses of IPTp in pregnancy [23]. In the central part of the country, quintuple and sextuple *pfdhfr/pfdhps* mutations combined were recently found in >90 % of *P. falciparum* infecting pregnant women at first ANC visit [24], and in the eastern part, more than a quarter of delivering women had evidence of active placental *P. falciparum* infection despite previous intake of ≥2 doses of SP [13]. In the latter region, IPT of school children with SP did not provide any benefit over placebo [25]. In the present study, the effectiveness of IPTp with SP in the western highland region of Fort Portal was estimated in a cross sectional study looking at effects on infection, anaemia, LBW, and preterm delivery as well as on the pattern of *pfdhfr/pfdhps* alleles.

## Methods

Fort Portal, located at 1500 m altitude, is a community of some 55,000 inhabitants and capital of the western Kabarole district, close to the border of DR Congo. Twenty years ago, an altitude of 1500 m in this district represented a threshold between hypo- and mesoendemic conditions [26]. The 2014 malaria indicator survey reports a prevalence of malaria parasites among children in the larger mid-Western region of 18 % [27]. The Holy Family Virika Hospital in Fort Portal is a private (Catholic Church) not-for-profit health facility and has a bed capacity of 155. It provides services to patients from Kabarole and surrounding districts and thereby supplements the governmental Fort Portal Regional Referral Hospital (330 beds). From February to December 2013, adult women attending Virika Hospital for delivery were asked to participate in the present cross-sectional study and recruited after informed written consent was obtained. The study protocol was reviewed and approved by the Higher Degrees, Research, and Ethics Committee, College of Health Sciences, Makerere University, Kampala, and by the Uganda National Council for Science and Technology.

All women were clinically examined. Fever was defined as an axillary temperature ≥37.5 °C. Obstetric and medical history was documented, as were socio-economic data. Participation in a programme on prevention of mother-to-child-transmission of HIV (PMTCT) was noted. Data on intake of SP or other anti-malarial drugs was verified on ANC cards. Venous peripheral blood was collected into EDTA; blood from the intervillous space was collected with a syringe containing EDTA following incision into the maternal surface of the placenta. Haemoglobin (Hb) was measured by a HemoCue photometer (Ångelholm, Sweden) and anaemia defined as Hb <11.5 g/dL increasing the threshold by +0.5 g/dL to account for altitude [28]. Birth weight and gestational age were assessed within 24 h after delivery. LBW was defined as a birth weight <2500 g and preterm delivery as gestational age <37 weeks applying the simple morphological Finnström score [29]. Malaria parasites were counted microscopically on Giemsa-stained thick blood films per 500 white blood cells for peripheral samples and per 100 high-power fields for placental samples. Following DNA extraction of peripheral blood samples (QIAmp, Qiagen, Germany), semi-nested PCR assays were performed for the diagnosis of *P. falciparum* and other species [30]. If not otherwise indicated, *P. falciparum* infection hereinafter refers to infection as detected by PCR. For *P. falciparum* resistance marker typing, restriction fragment length polymorphisms of PCR-generated amplicons identified mutations of *pfdhfr* (N51I, C59R, S108N, I164L) and *pfdhps* (A437G, K540E, A581G) [31]. Isolates with mixed alleles, i.e., both wildtype and mutation present, were considered mutant. Laboratory strains 3D7, HB3 and Dd2 served as controls.

Women were grouped into primiparae, parae 2 and 3, and multiparae (>3 previous deliveries). Geometric mean parasite densities (GMPDs) and 95 % confidence intervals (95 % CIs) were calculated. Continuous variables were compared between groups by t test, analysis of variance, Mann–Whitney U test, and Kruskal–Wallis test as applicable. Associations between categorical variables were identified by χ^2^ test or Fisher’s exact test, and odds ratios (ORs) were calculated. Multivariate logistic regression with stepwise removal of factors found to be not associated in multivariate analysis (*P* > 0.05) was used to identify independent predictors of *P. falciparum* infection. A *P* value of <0.05 was considered statistically significant.

## Results

Between February and December 2013, 915 delivering women were recruited and 945 babies (885 singles, 60 twins) were born, of whom 45 (4.9 %) were born dead. The characteristics of the 728 live singleton deliveries with available *P. falciparum* infection status by PCR are shown in Table 1. Data on chemoprevention was verified by checking ANC cards in 98.3 % (676/688) of these women. Most women originated from the local Kabarole district, and Mutooro ethnicity predominated. Travel to the hospital took a median of approximately 1 h. Almost one third benefited from a coverage programme for hospital costs. Unmarried mothers were common among primiparae but rare in multiparae who at the same time showed comparatively lower levels of formal education than primiparae. Multiparae showed reduced proxy parameters of socio-economic status, e.g., tap water or electricity on the premises.

**Table 1:** Characteristics of 728 women with live singleton deliveries

Parameter	All	Primiparae	Parae II and III	Multiparae (>III)	*P*
No. (%)	100 (728)	31.6 (229/725)	37.0 (268/725)	31.4 (228/725)	
Age (years; median, range)	25 (18–42)	20 (18–35)	24 (18–39)	30 (20–42)	<0.0001
Residence in Kabarole district (%, No.)	76.9 (498/648)	75.6 (155/205)	76.3 (187/245)	79.5 (155/195)	0.62
Travel distance to hospital (min., median, range; *n* = 648)	55 (2–360)	55 (3–360)	40 (5–300)	60 (2–300)	0.12
Mutooro ethnicity (%, No.)	58.9 (417/708)	53.2 (118/222)	61.4 (159/259)	61.9 (140/226)	0.10
Married (%, No.)	71.4 (517/724)	55.5 (126/227)	75.0 (201/268)	82.8 (188/227)	<0.0001
Proportion without formal education (%, No.)	6.9 (49/715)	5.3 (12/228)	3.0 (8/264)	13.1 (29/221)	<0.0001
Hospital cost coverage present (%, No.)	30.4 (207/682)	30.7 (66/215)	25.3 (65/257)	35.6 (74/208)	0.05
No. of people living in household (median, range; *n* = 706)	4.0 (1–22)	3.0 (1–12)	3.0 (1–11)	6.0 (2–22)	<0.0001
Tap water on premises (%, No.)	40.9 (296/724)	40.2 (92/229)	48.9 (131/268)	31.9 (72/226)	0.0006
Electricity on premises (%, No.)	27.5 (199/723)	26.6 (61/229)	37.8 (101/267)	15.9 (36/226)	<0.0001
Bed net in household (%, No.)	79.8 (574/719)	73.9 (167/226)	85.8 (230/268)	78.6 (176/224)	0.004
Antenatal care and malaria prevention					
No. of previous antenatal care visits (median, range; *n* = 657)	4.0 (0–9)	4.0 (0–7)	4.0 (0–9)	3.0 (0–8)	0.007
Proportion with ≤3 antenatal care visits (%, No.)	42.9 (282/657)	42.0 (87/207)	36.9 (89/241)	50.5 (105/208)	0.01
Participation in PMTCT programme (%, No.)	11.7 (85)	7.4 (17/229)	14.6 (39/268)	12.7 (29/228)	0.04
Referred for delivery (%, No.)	46.4 (334/720)	51.3 (117/228)	41.9 (111/265)	46.5 (105/226)	0.11
Slept under bed net last night (%, No.)	65.1 (436/670)	60.2 (127/211)	67.7 (170/251)	66.5 (137/206)	0.20
Malaria-preventive drug intake (%, No.)					
None	8.4 (58/688)	10.2 (22/216)	7.8 (20/258)	7.6 (16/211)	
IPTp 1 dose	25.6 (176/688)	30.6 (66/216)	20.9 (54/258)	26.1 (55/211)	
IPTp 2 doses	54.7 (376/688)	52.8 (114/216)	57.4 (148/258)	53.1 (112/211)	
Cotrimoxazole	7.8 (54/688)	3.2 (7/216)	11.2 (29/258)	8.5 (18/211)	
IPTp and cotrimaxazole	3.5 (24/688)	3.2 (7/216)	2.7 (7/258)	4.7 (10/211)	0.03
Week of gestation when taking IPTp dose 1 (median, range; *n* = 548)	24 (8–38)	23 (8–38)	22 (12–38)	24 (12–36)	0.02
Week of gestation when taking IPTp dose 2 (median, range; *n* = 365)	30 (20–40)	30 (21–39)	30 (20–40)	32 (20–39)	0.48
Received treatment for malaria episode during pregnancy (%, No.)	23.9 (164/685)	23.5 (51/217)	24.8 (63/254)	23.2 (49/211)	0.91
Clinical data					
Fever (%, No.)	1.0 (6/628)	1.0 (2/194)	0.9 (2/234)	1.0 (2/197)	0.98
Haemoglobin (g/dL, median, range; n = 692)	12.5 (4.0–16.7)	12.6 (6.1–16.5)	12.6 (4.0–16.7)	12.2 (4.5–16.6)	0.17
Anaemia (%, No.; Hb <11.5 g/dL)	28.9 (200/692)	27.9 (60/215)	26.5 (68/257)	33.2 (72/217)	0.25
Birth weight (g; median, range; n = 709)	3100 (800–4720)	3000 (1100–4200)	3120 (900–4500)	3160 (800–4720)	0.0001
Low birth weight (%, No.)	9.7 (69/709)	11.0 (24/219)	9.5 (25/263)	8.0 (18/225)	0.57
Gestational age (weeks; mean ± SD; *n* = 705)	38.5 ± 2.1	38.3 ± 2.0	38.6 ± 2.2	38.6 ± 2.2	0.19
Preterm delivery (%, No.)	11.9 (84/705)	15.9 (35/220)	8.8 (23/260)	11.7 (26/223)	0.06

Almost half of the women had been referred to the hospital for delivery, and 12 % participated in a PMTCT programme. The number of previous antenatal care visits was similar among primiparae and parae 2 and 3 but less in multiparae. Eighty percent of the women had taken at least one dose of IPTp (adding 3.5 % of those taking both IPTp and cotrimoxazole), and more than half had taken two doses. No chemoprophylaxis and daily cotrimoxazole were taken by each 8 % of the women. Cotrimoxazole intake was less common among primiparae whereas IPTp was non-significantly more frequent. Almost two thirds of women stated to have used a bed net in the previous night and one in four women reported to have received treatment for malaria during pregnancy, without differences by parity. Fever was rare. Anaemia (29 %) affected women of all parities. Birth weight increased with increasing parity and this was reflected by a respective trend towards less LBW. Preterm delivery was increased in primiparae (*P* = 0.03).

### Prevalence of *Plasmodium falciparum* infection

**Fig. 1:**
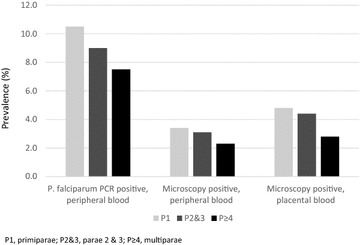
Prevalence (%) of *P. falciparum* according to parity

*Plasmodium falciparum* was detected in peripheral blood by PCR in 8.9 % (65/728) and by microscopy in 2.9 % (20/682). The geometric mean parasite density was 1986/µL (95 % CI, 602–6553). Placental parasitaemia was observed by microscopy in 4.0 % (27/676). 56.9 % (37/65) of the infections were submicroscopic, i.e., reflected by a positive PCR but negative microscopy result of peripheral or placental blood. Irrespective of diagnostic method, infection prevalence slightly and non-significantly declined with increasing parity (Fig. 1). Non-falciparum parasites were rare (seven *Plasmodium malariae*, one *Plasmodium ovale*) and not related to parity.

### Manifestation of *Plasmodium falciparum* infection

In all women available for analysis, *P. falciparum* infection (PCR) was associated with increased odds of stillbirth: it occurred in 4.3 % (31/716) of non-infected and in 10.7 % (8/75) of infected mothers (OR, 2.64; 95 % CI, 1.1–6.3; *P* = 0.02). This association was pronounced for infections detected by placental microscopy [4.3 % (35/808) vs. 14.7 % (5/34); OR, 3.81; 95 % CI, 1.1–10.8; *P* = 0.02] but non-significant for submicroscopic infections (OR, 2.33; 95 % CI, 0.6–7.1; *P* = 0.12).

Among women with live singleton delivery, 13 children died within 24 h of delivery. In 30.8 % (4/13) of these, maternal *P. falciparum* infection had been observed as compared to 8.5 % (61/715) among mothers of surviving children (OR, 4.77; 95 % CI, 1.0–17.6; *P* = 0.02). Moreover, *P. falciparum* infection was associated with each more than doubled odds of anaemia, LBW and preterm delivery (Table 2). Correspondingly, in infected (PCR) as compared to non-infected mothers, median Hb concentration, median birth weight, and mean gestational age were reduced by 1.0 g/dL (*P* < 0.0001), 130 g (*P* = 0.03), and 1 week (*P* = 0.01), respectively. Anaemia and LBW were not increased in women with submicroscopic infection, but preterm delivery showed a respective trend (Table 2).

**Table 2:** Low birth weight, preterm delivery, and maternal anaemia according to *Plasmodium falciparum* infection

Parameter	Low birth weight			Preterm delivery			Anaemia		
	% (n/n)	OR (95 % CI)	*P*	% (n/n)	OR (95 % CI)	*P*	% (n/n)	OR (95 % CI)	*P*
*P. falciparum* PCR, peripheral blood									
Negative	9.0 (58/648)	1		10.8 (69/641)	1		27.0 (170/629)	1	
Positive	18.0 (11/61)	2.24 (1.0–4.7)	0.02	23.4 (15/64)	2.54 (1.3–5.0)	0.003	47.6 (30/63)	2.45 (1.4–4.3)	0.0006
*P. falciparum* infection									
None	9.0 (56/624)	1		10.0 (62/617)	1		26.9 (169/628)	1	
Submicroscopic	8.6 (3/35)	0.95 (0.2–3.2)	1.0	19.4 (7/36)	2.16 (0.8–5.3)	0.09	32.4 (12/37)	1.30 (0.6–2.8)	0.46
Microscopic	30.8 (8/26)	4.51 (1.7–11.6)	0.002	28.6 (8/28)	3.58 (1.4–9.0)	0.007	70.4 (19/27)	6.45 (2.6–16.4)	<0.0001

*OR* odds ratio, *95* *% CI* 95 % confidence interval

### Impact of intermittent preventive treatment

IPTp had no significant influence on the presence of *P. falciparum* infection. Nor was it associated with reductions in anaemia, LBW or preterm delivery (Table 3). Correspondingly, *P. falciparum* infection in women using IPTp was associated with reductions in median Hb concentration, median birth weight, and mean gestational age of 0.85 g/dL (*P* = 0.0008), 207 g (*P* = 0.004), and 1.1 weeks (*P* = 0.0003), respectively. Stratification by parity did not change these overall findings (Table 4). Moreover, infection prevalence was not reduced in women having taken two doses of IPTp (10.9 %, 41/376) as compared to one dose (6.8 %, 12/176). Timing of last IPTp intake (weeks ago) and infection were not associated (*P* = 0.93). In women on cotrimoxazole or IPTp *plus* cotrimoxazole, infection prevalence was substantially reduced but not significantly so (Table 3).

**Table 3:** Prevalence of *P. falciparum* infection according to the reported use of IPTp and/or cotrimoxazole

Parameter	None	IPTp	CTX	IPTp + CTX	No data
*P. falciparum* infection (PCR)					
%, No.	13.8 (8/58)	9.6 (53/552)	3.7 (2/54)	0 (0/24)	5.0 (2/40)
OR (95 % CI), *P*	Ref.	0.66 (0.3–1.6), *P* = 0.31	0.24 (0.0–1.3), *P* = 0.10	0 (0.0–1.4), *P* = 0.10	0.33 (0.0–1.8), *P* = 0.19
Placental parasitaemia					
%, No.	4.0 (2/50)	4.7 (24/515)	2.0 (1/51)	0 (0/23)	0 (0/37)
OR (95 % CI), *P*	Ref.	1.17 (0.3–10.5), *P* = 1.0	0.48 (0.0–9.6), *P* = 0.62	0.0 (0.0–11.7), *P* = 1.0	0.0 (0.0–7.2), *P* = 0.51
Maternal anaemia					
%, No.	26.4 (14/53)	27.4 (145/529)	38.0 (19/50)	26.1 (6/23)	43.2 (16/37)
OR (95 % CI), *P*	Ref.	1.05 (0.5–2.1), *P* = 0.88	1.71 (0.7–4.3), *P* = 0.21	0.98 (0.3–3.3), *P* = 0.98	2.12 (0.8–5.7), *P* = 0.10
Low birth weight					
%, No.	13.8 (8/58)	9.5 (51/535)	9.3 (5/54)	8.3 (2/24)	7.9 (3/38)
OR (95 % CI), *P*	Ref.	0.66 (0.3–1.6), *P* = 0.3	0.64 (0.2–2.4), *P* = 0.45	0.57 (0.1–3.2), *P* = 0.72	0.54 (0.1–2.5), *P* = 0.52
Preterm delivery					
%, No.	10.7 (6/56)	11.4 (61/534)	17.0 (9/53)	13.0 (3/23)	12.8 (5/39)
OR (95 % CI), *P*	Ref.	1.07 (0.4–3.2), *P* = 0.87	1.70 (0.5–6.3); *P* = 0.34	1.25 (0.2–6.6), *P* = 0.71	1.23 (0.3–5.3), *P* = 0.76

*IPTP* intermittent preventive treatment in pregnancy, *CTX* cotrimoxazole, *OR* odds ratio, *95* *% CI* 95 % confidence interval

**Table 4:** Prevalence of *P. falciparum* infection according to the reported use of IPTp and/or cotrimoxazole, and parity

Parameter	Primiparae					Parae 2 and 3					Multiparae				
	None	IPTp	CTX	IPTp + CTX	No data	None	IPTp	CTX	IPTp + CTX	No data	None	IPTp	CTX	IPTp + CTX	No data
*P. falciparum* infection (PCR; %; No.)	13.6 (3/22)	11.1 (20/180)	0 (0/7)	0 (0/7)	7.7 (1/13)	20.0 (4/20)	8.9 (18/202)	6.9 (2/29)	0 (0/7)	0 (0/10)	6.3 (1/16)	9.0 (15/167)	0 (0/18)	0 (0/10)	5.9 (1/17)
Placental parasitaemia (%, No.)	0 (0/18)	6.1 (10/164)	0 (0/7)	0 (0/7)	0 (0/13)	11.1 (2/18)	4.2 (8/189)	3.7 (1/27)	0 (0/7)	0 (0/10)	0 (0/14)	3.8 (6/159)	0 (0/17)	0 (0/9)	0 (0/14)
Maternal anaemia (%, No.)	21.1 (4/19)	27.2 (46/169)	42.9 (3/7)	28.6 (2/7)	38.5 (5/13)	16.7 (3/18)	26.2 (51/195)	33.3 (9/27)	14.3 (1/7)	40.0 (4/10)	43.8 (7/16)	29.6 (48/162)	43.8 (7/16)	33.3 (3/9)	50.0 (7/14)
Low birth weight (%, No.)	13.6 (3/22)	11.6 (20/172)	0 (0/7)	0 (0/7)	9.1 (1/11)	10.0 (2/20)	8.1 (16/197)	10.3 (3/29)	28.6 (2/7)	20.0 (2/10)	18.8 (3/16)	7.9 (13/164)	11.1 (2/18)	0 (0/10)	0 (0/17)
Preterm delivery (%, No.)	4.5 (1/22)	16.3 (28/172)	28.6 (2/7)	28.6 (2/7)	16.7 (2/12)	15.8 (3/19)	7.7 (15/196)	10.3 (3/29)	0 (0/6)	20.0 (2/10)	13.3 (2/15)	11.0 (18/164)	23.5 (4/17)	10.0 (1/10)	5.9 (1/17)

No significant differences between women with and without chemoprevention were observed

*IPTP* intermittent preventive treatment in pregnancy, *CTX* cotrimoxazole

### Factors associated with *Plasmodium falciparum* infection

In univariate analysis, the odds of *P. falciparum* infection declined with age, presence of electricity in the household, bed net ownership, bed net usage in the preceding night, and Mutooro ethnicity, and increased with referral to hospital for delivery and travel distance to the hospital. In multivariate analysis, referral and travel distance proved to be independent predictors of infection while age and household electricity were negatively associated (Table 5). Further, partly proximate, factors were not associated with infection, including parity, educational level, occupation, district of residence, number of people living in the household, other proxy indicator of socio-economic status, number of antenatal care visits and participation in the PMTCT programme. In the above multivariate model, IPTp did not significantly influence the odds of *P. falciparum* infection (aOR, 0.59; 95 % CI, 0.25–1.37; *P* = 0.22).

**Table 5:** Factors associated with *P. falciparum* infection

Parameter	No.	% Infected	Univariate analysis		Multivariate analysis^a^	
			OR (95 % CI)	*P*	aOR (95 % CI)	*P*
Age (years)	728	n.a.	0.93 (0.88–0.98)	0.005	0.94 (0.89–0.99)	0.02
Referred for delivery						
No	386	5.2	1			
Yes	334	13.2	2.78 (1.55–5.0)	0.0002	2.11 (1.17–3.81)	0.01
Travel distance to hospital						
≤1 h	477	7.3	1			
>1 h	171	15.2	2.26 (1.27–4.02)	0.003	1.80 (1.02–3.17)	0.04
Electricity on the premises						
No	524	11.1	1			
Yes	199	3.5	0.29 (0.11–0.66)	0.002	0.38 (0.17–0.86)	0.02
Used bet net last night						
No	234	12.8	1			
Yes	436	6.9	0.50 (0.29–0.88)	0.01		
Bed net ownership						
No	145	13.8	1			
Yes	574	7.8	0.53 (0.29–0.97)	0.03		
Ethnic group						
Mutooro	417	7	1			
Else	291	11.3	1.71 (0.98–2.98)	0.04		

*OR* odds ratio, *aOR* adjusted OR, *95* *% CI* 95 % confidence interval

^a^Adjusted for other factors significantly associated with outcome; n = 643

### Molecular markers of drug resistance

**Fig. 2:**
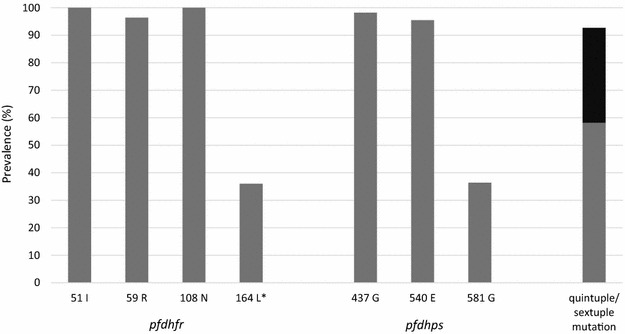
Prevalence (%) of *pfdhfr* and *pfdhps* mutations in 55 *P. falciparum* isolates from western Uganda. *Asterisk* n = 50. Sextuple mutation is displayed in *black*

Typing of essential *pfdhfr* and *pfdhps* alleles was successful for 55 (85 %) isolates. Mutant alleles were found in ≥95 % each for *pfdhfr* codons 51, 59, and 108 as well as for *pfdhps* codons 437 and 540 (Fig. 2). In consequence, *pfdhfr/pfdhps* quintuple and sextuple mutations were observed in 93 % (51/55) of the isolates. The high resistance alleles *pfdhfr* 164L and *pfdhps* 581G occurred each in 36 % (18/50; 20/55); *pfdhps* 581G in 42 % (8/19) occurred together with *pfdhfr* 164L. *pfdhps* 581G was associated with increased placental parasitaemia as compared to wildtype parasites, both in the overall group [GMPD, 22/100 high power fields; 95 % CI, 7–70 vs. 4 (2–7), *P* = 0.01] and among women who had been taking IPTp [25 (7–87) vs. 4 (2–9), *P* = 0.02].

## Discussion

In this highland area of western Uganda, though malaria in pregnancy is comparatively rare, it substantially contributes to mortality and morbidity including stillbirth, early neonatal death, anaemia, LBW and preterm delivery. IPTp, recommended for the prevention of malaria and its consequences, did not show a beneficial effect. One very likely reason is the vast predominance of highly resistant strains of *P. falciparum*.

The protective efficacy of IPTp with SP against placental malaria has been estimated as roughly 50 % in areas of low to moderate SP resistance and over a decade ago [32]. As a major limitation, the present cross-sectional study lacked power to display an only modest impact of IPTp. Considering the given group sizes and prevalence, the study was powered to detect an effect of IPTp on *P. falciparum* infection at a magnitude of >75 % reduction. Nevertheless, infection prevalence was actually higher in women having taken two as compared to one dose of SP, and placental parasitaemia, anaemia, and preterm delivery were slightly more common in women having taken IPTp as compared to women without drug-based prevention. A further limitation refers to the validity of reported IPTp use on which the current analysis is based. However, in Tanzania, reported use and detection of plasma sulfa levels matched closely [11] suggesting that reported use is not unreliable per se. It appears, therefore, justifiable to state that IPTp did not fulfil its purpose. Because HIV negativity has yet to be confirmed by molecular means in the study group, participation in a PMTCT programme was considered as a proxy indicator of HIV status. PMTCT participation and cotrimoxazole intake overlapped largely. It is, therefore, not possible to make firm statements on an impact of HIV infection on *P. falciparum* infection or pregnancy outcomes but the analyses do not provide evidence for respective effects. Also, data on some potentially interfering factors were not available, e.g., syphilis. This should be kept in mind when interpreting the data.

*Plasmodium falciparum* infection was detected by microscopy of peripheral and placental blood films in only 3 and 4 %, respectively. Peripheral blood microscopy is notoriously insensitive in pregnant women whereas the sensitivity of PCR assays in detecting placental parasitaemia exceeds 95 % [4]. Submicroscopic infections in pregnancy are common and may contribute substantially to maternal and foetal morbidity [3, 4, 33]. In the present study, however, they did not associate with delivery outcomes, with the potential exemption of a borderline increased risk of preterm delivery. In contrast, malaria in pregnancy *per se* greatly increased the odds of stillbirth, early neonatal death, anaemia, LBW and preterm delivery. Even if comparatively rare at 9 %, this emphasizes the need for effective prevention of malaria in pregnancy in the study area. Peripheral residence and lacking electricity predicted *P. falciparum* infection illustrating the poverty-related nature of malaria. Bed net use, even though not significantly associated in multivariate analysis, was stated by almost two thirds of women and roughly halved the odds of infection. This highlights the opportunity and benefits of increasing bed net use among pregnant women in the study area.

Beyond statistical significance, *P. falciparum* prevalence was greatly reduced in women on cotrimoxazole and absent in those taking both cotrimoxazole and IPTp. This accords with findings from Malawi [34]. A slight superiority of daily cotrimoxazole over IPTp with SP in HIV-infected pregnant women was also observed in Togo [35] whereas the regimens had similar effects in Uganda [36] and Zambia [37]. Data of the present study, although comprising small numbers only, support the policy of using daily cotrimoxazole for malaria prevention in HIV-infected pregnant women instead of SP-IPTp. As a matter of fact, IPTp with SP is not recommended in HIV-infected women receiving daily cotrimoxazole because of additive sulfa toxicity [38].

Resistance marker typing in peripheral blood is reasonably representative of *P. falciparum* infecting pregnant women [39]. In the present study, *pfdhfr/pfdhps* quintuple mutants were close to fixation, and sextuple mutants and the high-grade resistance allele *pfdhfr* 164L occurred in one third of isolates. This accords with recent data from pregnant women in central Uganda [24], with one notable exception: there, only few parasites exhibited the *pfdhps* 581G mutation even though the proportion increased after IPTp. Consequently, in central Uganda, sextuple mutants made up less than a third of the proportion observed in Fort Portal. Against a background of intense antifolate resistance, this indicates an even higher degree in the present study area. The *pfdhps* 581G mutation (making up the sextuple mutant) has been considered to halve the protective period provided by a curative dose of SP [14] and to be associated with reduced birth weights and an increased risk of patent infection among mothers taking IPTp [16, 22]. In the present study, it was associated with increased placental parasite density, which accords with recent findings from Malawi [22]. Recent work has shown that this mutation has occurred multiple times on local *pfdhps* double-mutant backgrounds [40] and emerges in East Africa [41–45]. Moreover, in the present study, the *pfdhfr* 164L mutation occurred in 36 %, which is the highest figure reported from Africa [6, 18]. A previous study from southwestern Uganda found this high-grade resistance allele in 4 % and 14 % [43]. Even though the molecular data suggest intense SP resistance in the study area, the actual meaning for IPTp is not clear-cut. In central Uganda, despite >98 % *pfdhfr/pfdhps* quintuple mutants (but at a low prevalence of *pfdhps* 581G), 50 % of initially *P. falciparum* infected pregnant women became negative after one or two rounds of IPTp [24]. A current meta-analysis suggests a prevalence threshold of *pfdhps* 581G at which IPTp no longer protects against LBW of >10.1 % [21]. WHO recently considered the discontinuation of IPTp with SP in case of *P. falciparum* population prevalences of *pfdhps* 540E >95 % and *pfdhps* 581G >10 % [20]. In the present study, these thresholds are basically met (*pfdhps* 540E, 94.5 %; 581G, 36 %).

What then could be alternatives for the prevention of malaria in pregnancy in the study area? IPTp with mefloquine has disappointed expectations [46] and is not recommended [20]. WHO advises that in areas where IPTp-SP is discontinued because of resistance, access of pregnant women to long-lasting insecticide treated nets and to prompt diagnosis and effective treatment should be ensured. In the study area, bed net use can in fact be increased. Diagnosis, preferentially with a sensitive antigen capture test [4], preceding case management requires an easily accessible health system and an alert population. The same applies to the concept of intermittent screening and treatment [47]. Moreover, for both approaches, the issue of asymptomatic but still deleterious infections remains unsolved. Artemisinin-based combination therapy (ACT) is recommended for the treatment of malaria in pregnancy [38] but their use in IPTp has not been evaluated and may be complicated by the necessity of a 3-day regimen.

## Conclusion

Malaria in pregnancy in the area of Fort Portal, western Uganda, is comparatively rare but contributes significantly to stillbirth, anaemia, LBW, and preterm delivery. The molecular markers of *P. falciparum* show a very high degree of SP resistance, and reach the threshold set by WHO for the discontinuation of IPTp with SP. In line with that, IPTp with SP did not provide an observable benefit. Alternative approaches for the prevention of malaria in pregnancy are urgently needed.
